# Supplementary material for: Critical role of slags in pitting corrosion of additively manufactured stainless steel in simulated seawater
Source: Nat Commun. 2024 Jan 29;15:867. doi: 10.1038/s41467-024-45120-6 (PMC10825210; doi:10.1038/s41467-024-45120-6)
Supplement: Supplementary file 1 — Supplementary Information [file 41467_2024_45120_MOESM1_ESM.pdf]

Supplementary Information for

Critical role of slags in pitting corrosion of additively manufactured stainless steel in simulated seawater

Shohini Sen-Britain[1], Seongkoo Cho[1], ShinYoung Kang[1], Zhen Qi[1], Saad Khairallah[1], Debra Rosas[1], Vanna Som[1], Tian T. Li[1], S. Roger Qiu[1], Y. Morris Wang[2], Brandon C. Wood[1], Thomas Voisin[1§]

[1]Materials Science Division, Lawrence Livermore National Laboratory, Livermore, CA
[2]Department of Materials Science and Engineering, University of California Los Angeles, Los Angeles, CA

[§]corresponding author: voisin2@llnl.gov

SUPPLEMENTARY TABLES

Supplementary Table 1: Slags compositions in at. % measured by scanning transmission electron microscopy energy dispersive X-ray spectroscopy.

	Mn	Si	O	Cr	Al	Fe
**Type I top**	16.2	17.3	61.9	4.6	1.7	0.5
**Type I side**	10.5	15.5	67.0	7.0	?	0.5
**Type II**	16.8	17.8	58.4	7.1	1.2	0.5

Supplementary Table 2: Spherical particles compositions in slags in at. % measured by scanning transmission electron microscopy energy dispersive X-ray spectroscopy.

	Fe	Cr	Ni
**Type I top**	60.0	33.3	6.7
**Type I side**	68.5	29.8	1.7
**Type II**	72.2	25	2.8

SUPPLEMENTARY FIGURES

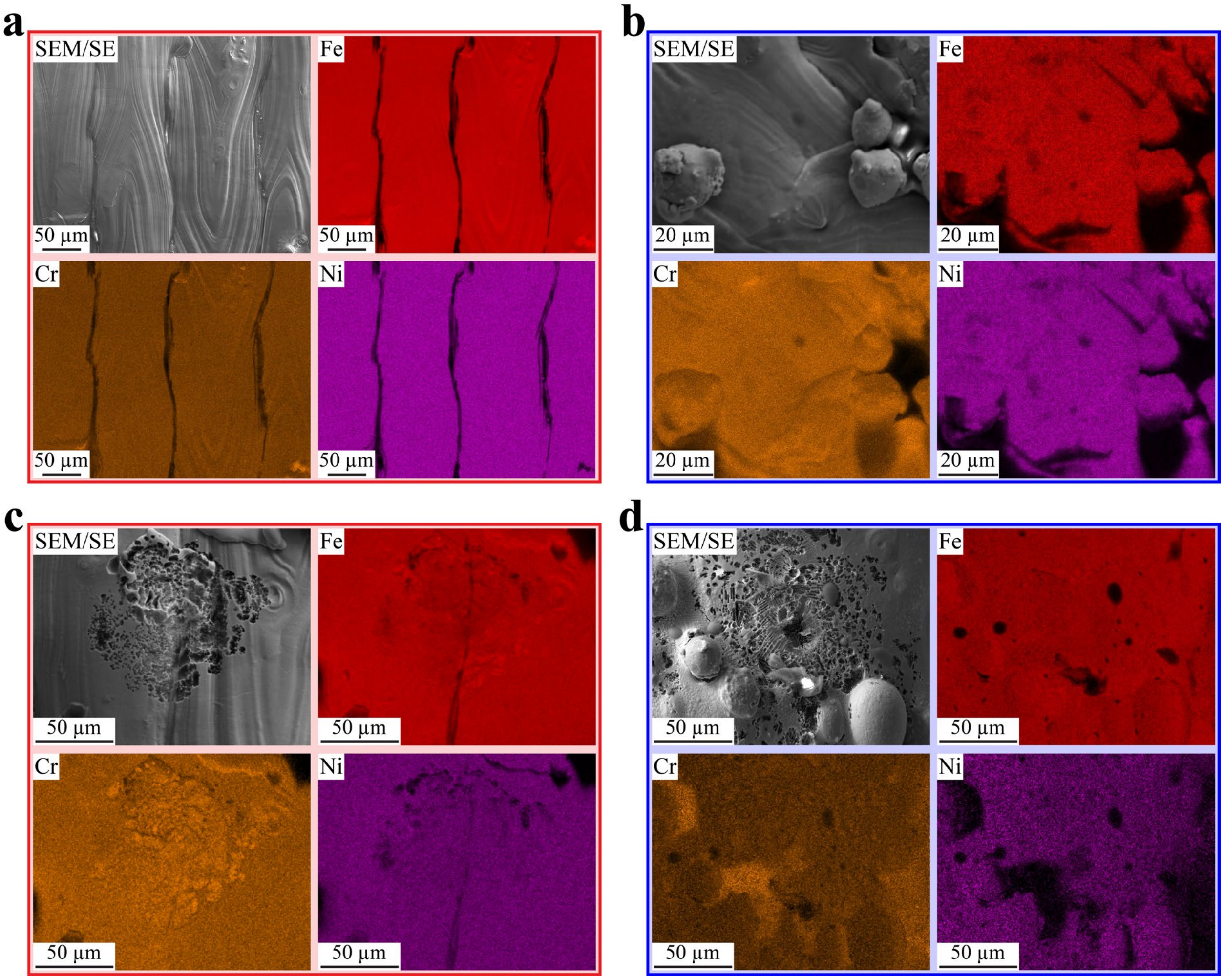

Supplementary Figure 1: Scanning electron microscopy energy dispersive X-ray spectroscopy (SEM/EDS) characterization of slags on as-built surfaces before and after pitting. This figure supports Figures 1 and 2 in the main manuscript. **a**. and **b**. top and side surfaces, respectively, before corrosion testing. **c**. and **d**. top and side surfaces, respectively, after pitting.

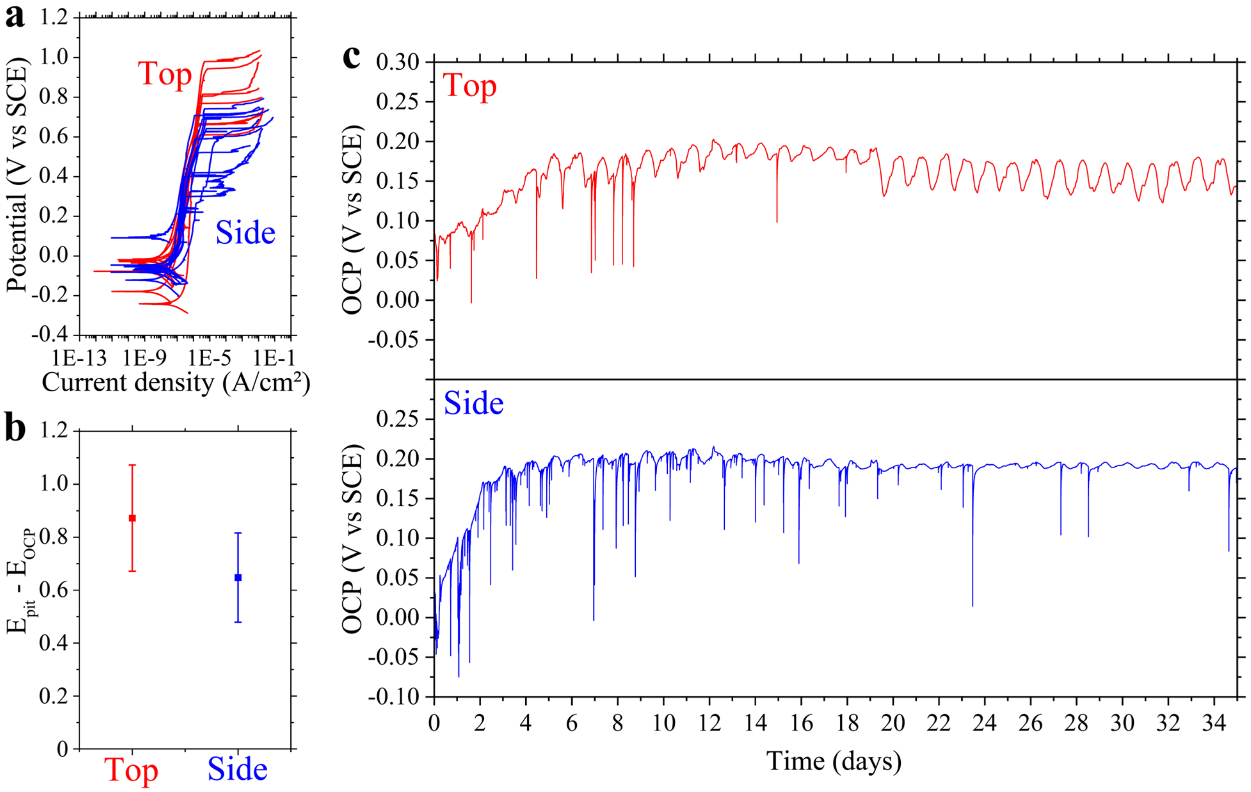

Supplementary Figure 2: Corrosion properties of the laser powder bed fusion (LPBF) 316L stainless steel as-built top and side surfaces in 0.6M NaCl solution. **a**. Potentiodynamic polarization curves after testing in 0.6M NaCl solution. 10 tests were conducted for each surface orientation. b. Average $E_{Pit} - E_{OCP}$ extracted from **a**. Error bars are the standard deviations. **c**. Open circuit potential (OCP) curves after 35 days of immersion in 0.6M NaCl for both top and side surfaces.

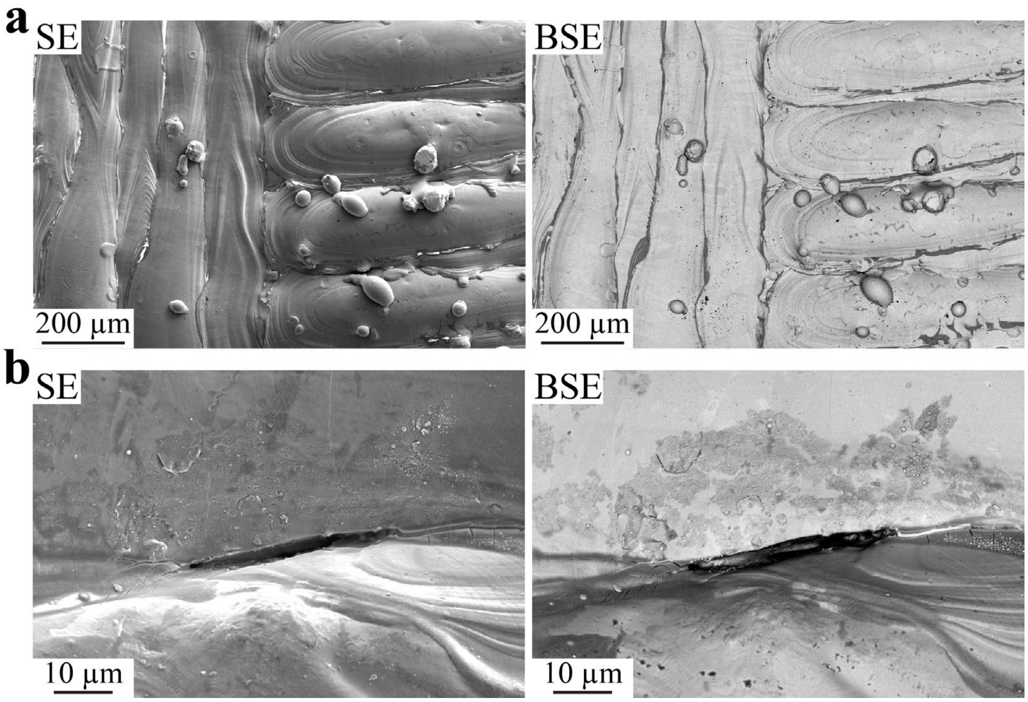

Supplementary Figure 3: Scanning electron microscopy images of as-built top surface after 35 days of open circuit potential (OCP) testing. **a**. Low magnification highlighting the inexistence of large pits. **b**. Higher magnification showing a pit at a slag. Slags can be distinguished by their darker contrast, especially on the BSE images, and the presence of small metallic inclusions. SE stands for secondary electrons, BSE for backscattered electrons.

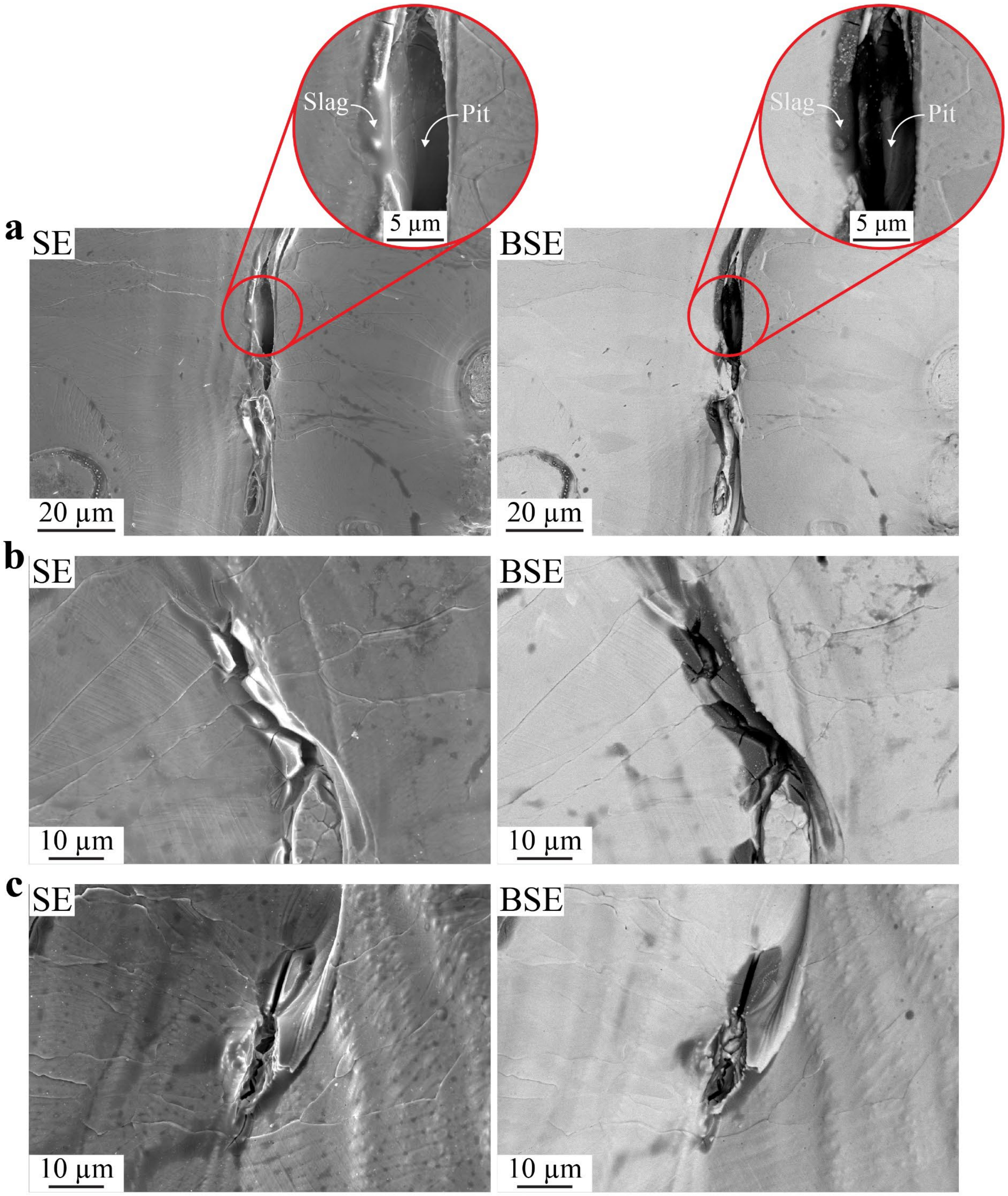

Supplementary Figure 4: Scanning electron microscopy images of as-built side surfaces after 35 days of open circuit potential (OCP) testing. **a**. to **c.** are various locations with pits found at slags. Slags can be distinguished by their darker contrast, especially on the BSE images, and the presence of small metallic inclusions. SE stands for secondary electrons, BSE for backscattered electrons.

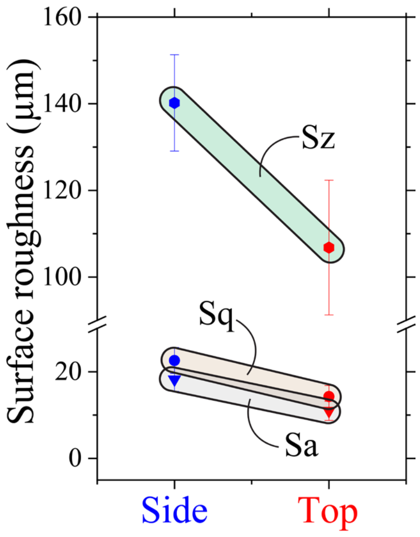

Supplementary Figure 5: Surface roughness of as-built surfaces. Error bars are standard deviations. Sz stands for maximum height, Sq for root mean square height, and Sa for arithmetical mean height.

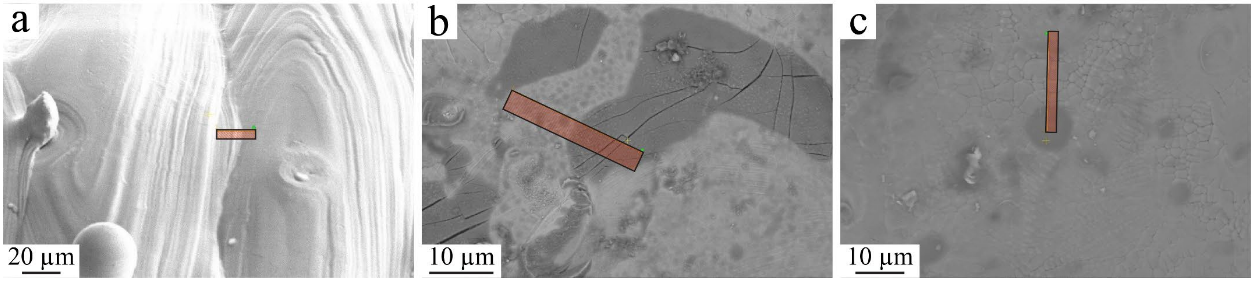

Supplementary Figure 6: Scanning electron microscopy images using secondary electrons showing the locations (red rectangles) of the focus ion beam (FIB) lift-out samples characterized by transmission electron microscopy in **a.** and **b.** Figure 4, and **c.** Figure 6, of the main manuscript.

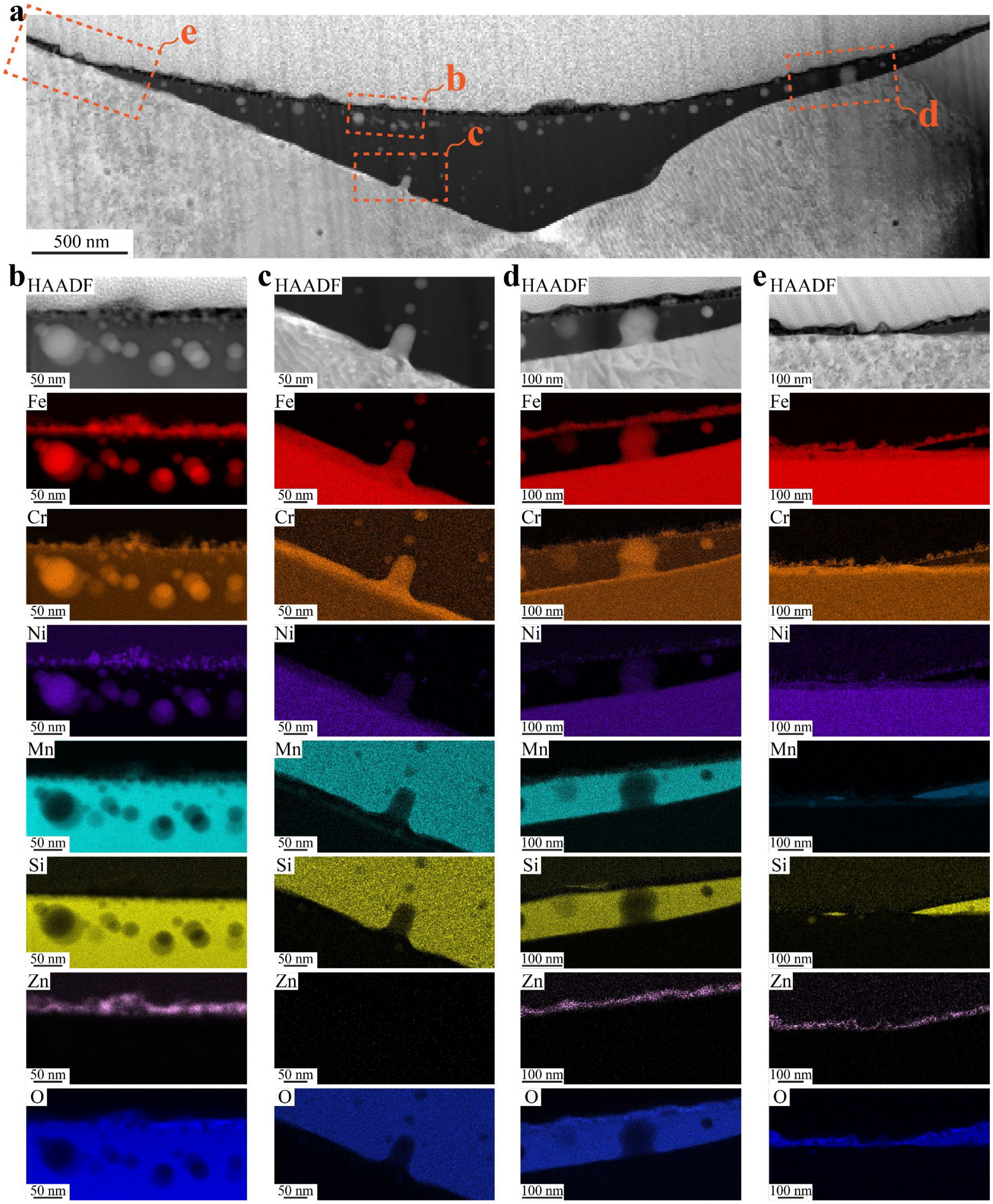

Supplementary Figure 7: Additional characterization by scanning transmission electron microscopy energy dispersive X-ray spectroscopy (STEM/EDS) of the Top I top slag presented in Figure 4a. and b. in the main manuscript. **a**. STEM high angle annular dark field (HAADF) image of the slag cross-sectioned. **b**. **to e**. STEM/EDS maps of the regions highlighted by red dashed rectangles in **a**.

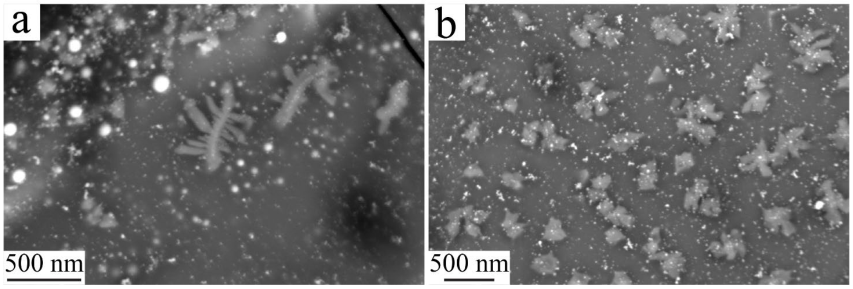

Supplementary Figure 8: Secondary electron scanning electron microscopy images taken at 5 keV and 0.4 nA to show irregularly shaped inclusions just below the surface inside Type I top slags.

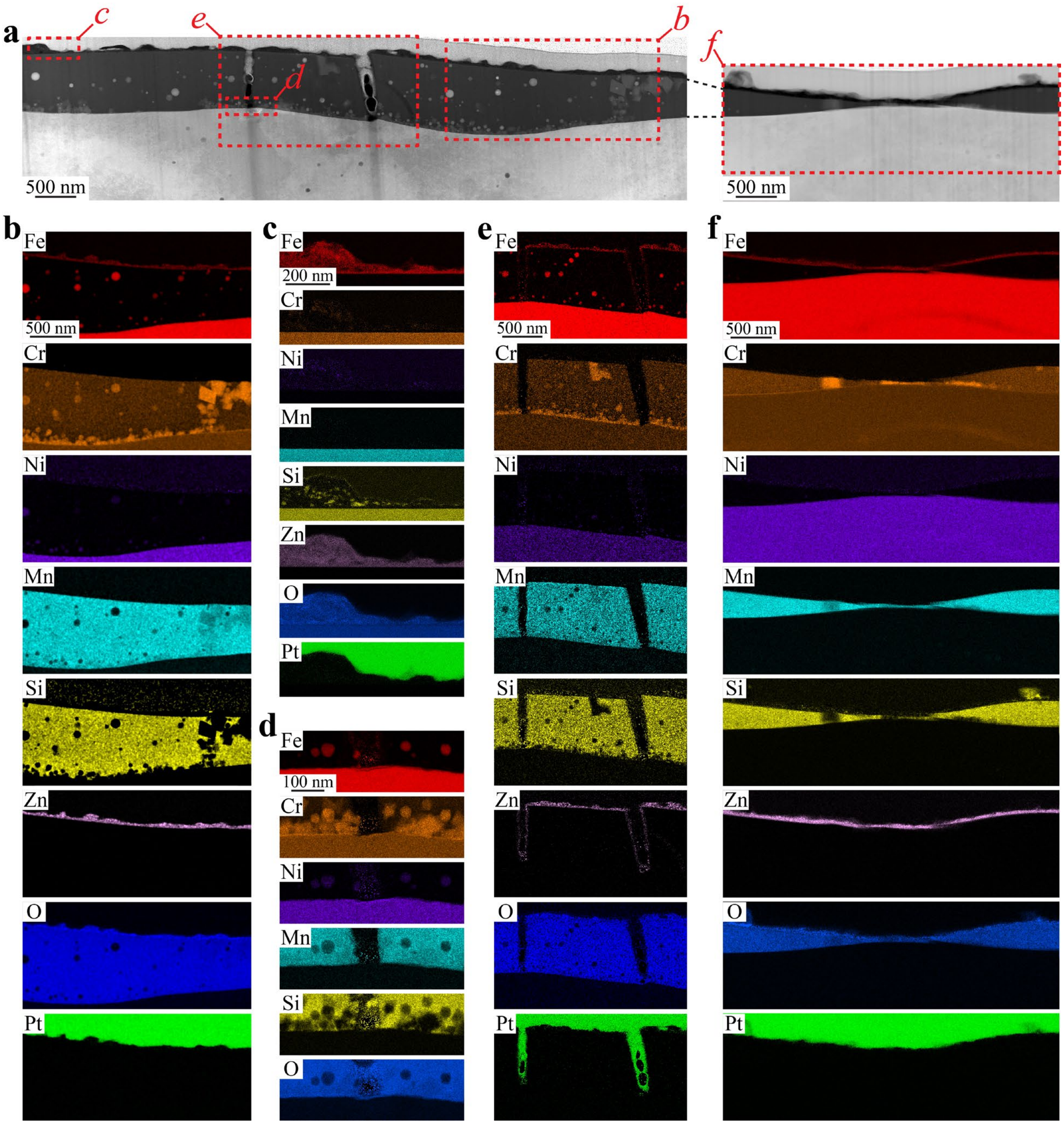

Supplementary Figure 9: Additional characterization by scanning transmission electron microscopy energy dispersive X-ray spectroscopy (STEM/EDS) of the Top I side slag presented in Figure 4c. and d. in the main manuscript. **a**. STEM high angle annular dark field (HAADF) image of the slag cross-sectioned. **b**. **to f**. STEM/EDS maps of the regions highlighted by red dashed rectangles in **a**.

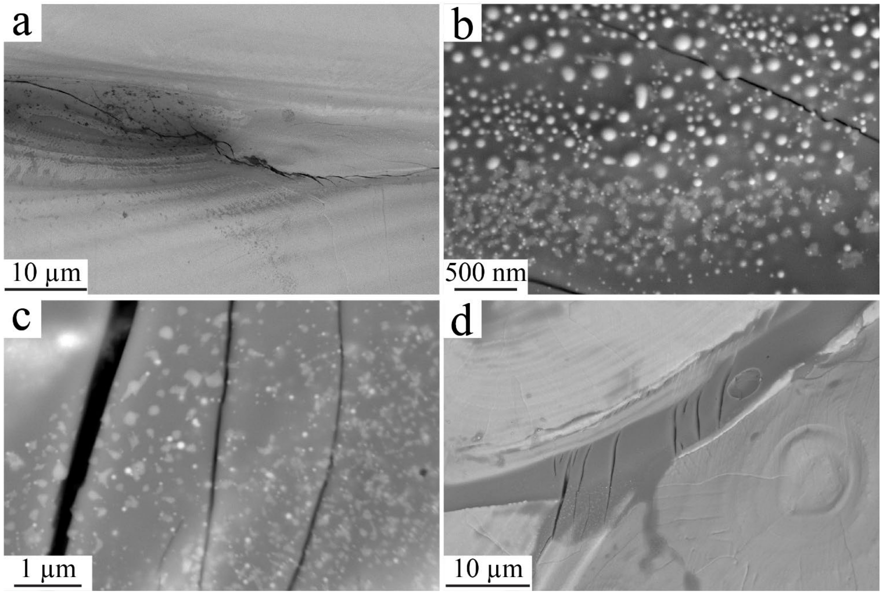

Supplementary Figure 10: Secondary electron scanning electron microscopy images of cracks in Type I **a. - b.** top and **c. - d.** side slags.

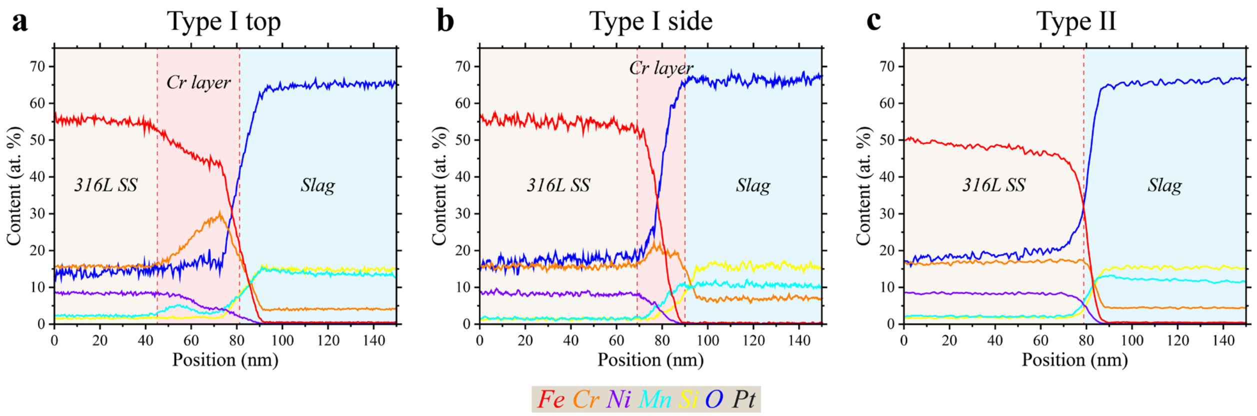

Supplementary Figure 11: Scanning transmission electron microscopy energy dispersive X-ray spectroscopy (STEM/EDS) line analysis of the metal/slag interface for **a.** and **b.** Type I and **c.** Type II slags.

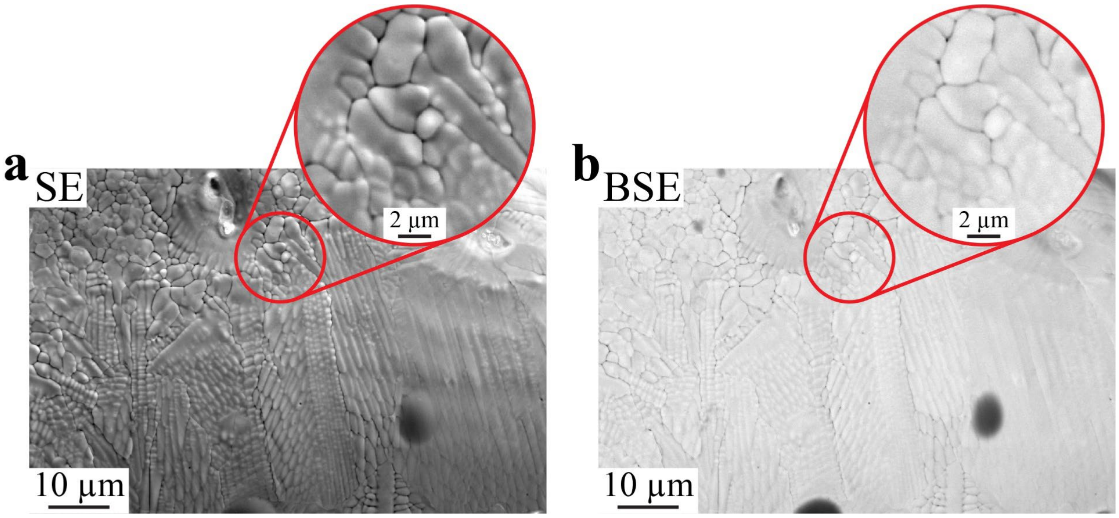

Supplementary Figure 12: Scanning electron microscopy images taken in the same region of an as-built surface in the vicinity of Type II slags before corrosion. **a.** Image obtained with secondary electrons. **b.** Image obtained with backscattered electrons.

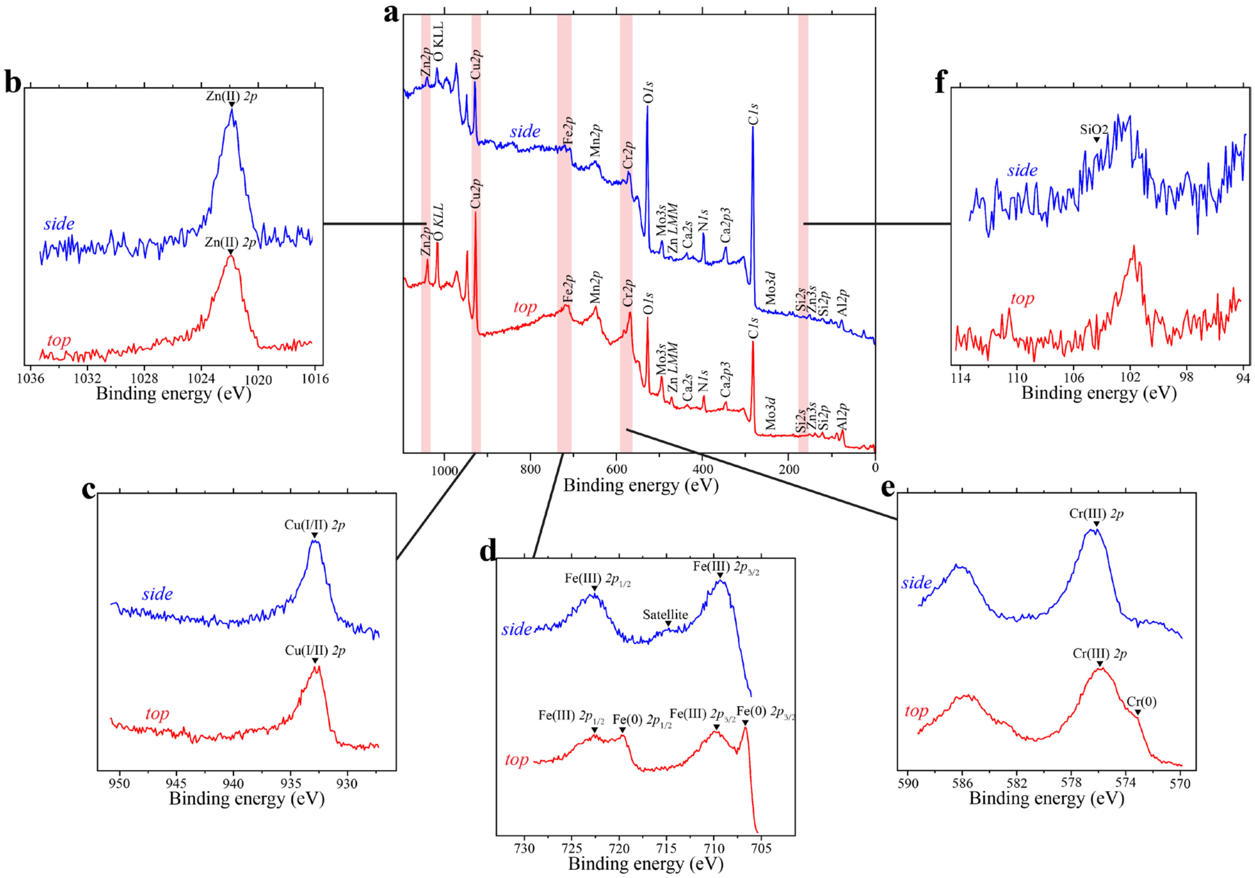

Supplementary Figure 13: X-ray photoelectron spectroscopy (XPS) performed on as-built top and side surfaces. **a.** Survey spectra collected for side (blue) and top (red) surface. **b.** to **f.** High resolution spectra collected for Zn, Cu, Fe, Cr, and Si.

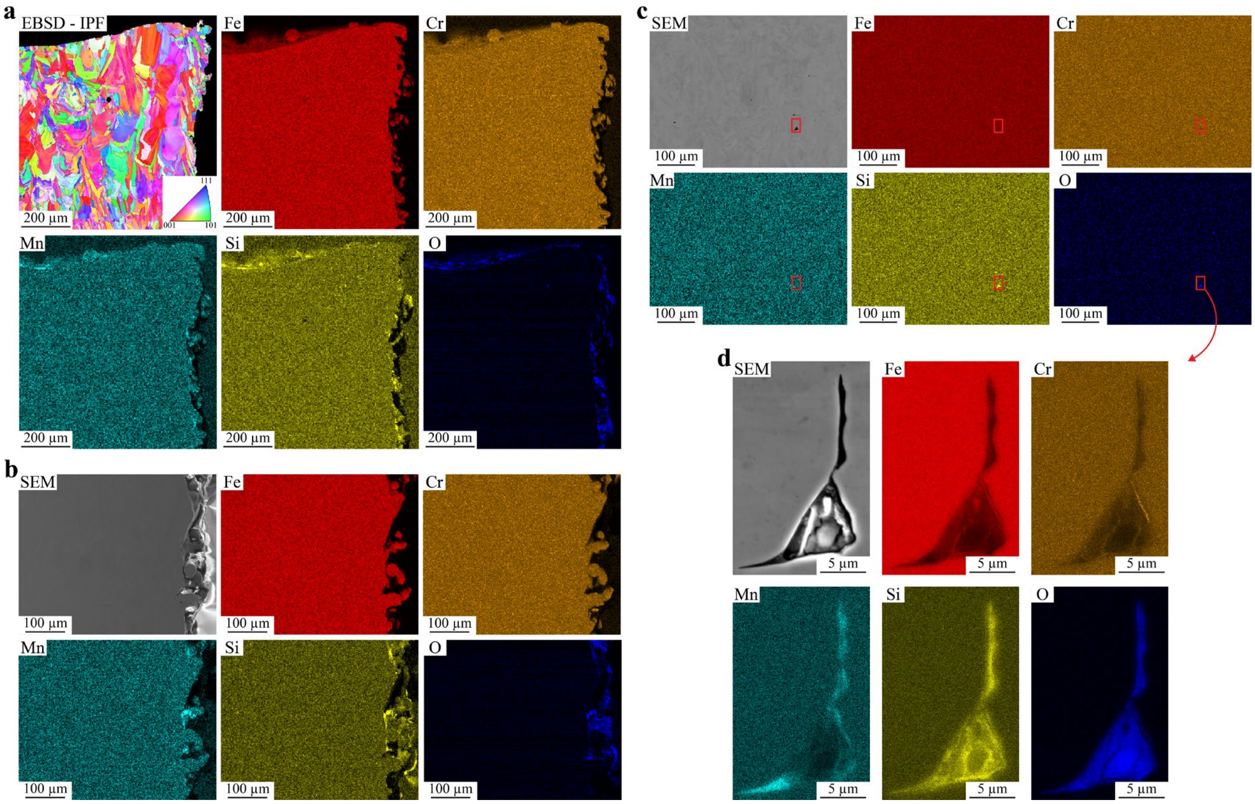

Supplementary Figure 14: Scanning electron microscopy energy dispersive X-ray spectroscopy (SEM/EDS) investigation showing that slags are mostly located at as-surfaces. **a.** SEM electron backscattered diffraction (EBSD) and EDS elemental maps of the corner of a part. The build direction is upward. Mn, Si, and O maps show that slags are all at the as-built surfaces. **b.** is a higher magnification SEM/EDS near the side surface. **c.** is a SEM/EDS taken inside the part, away from the surfaces. One slag can be seen in the red rectangle and is shown at higher magnification in **d.**

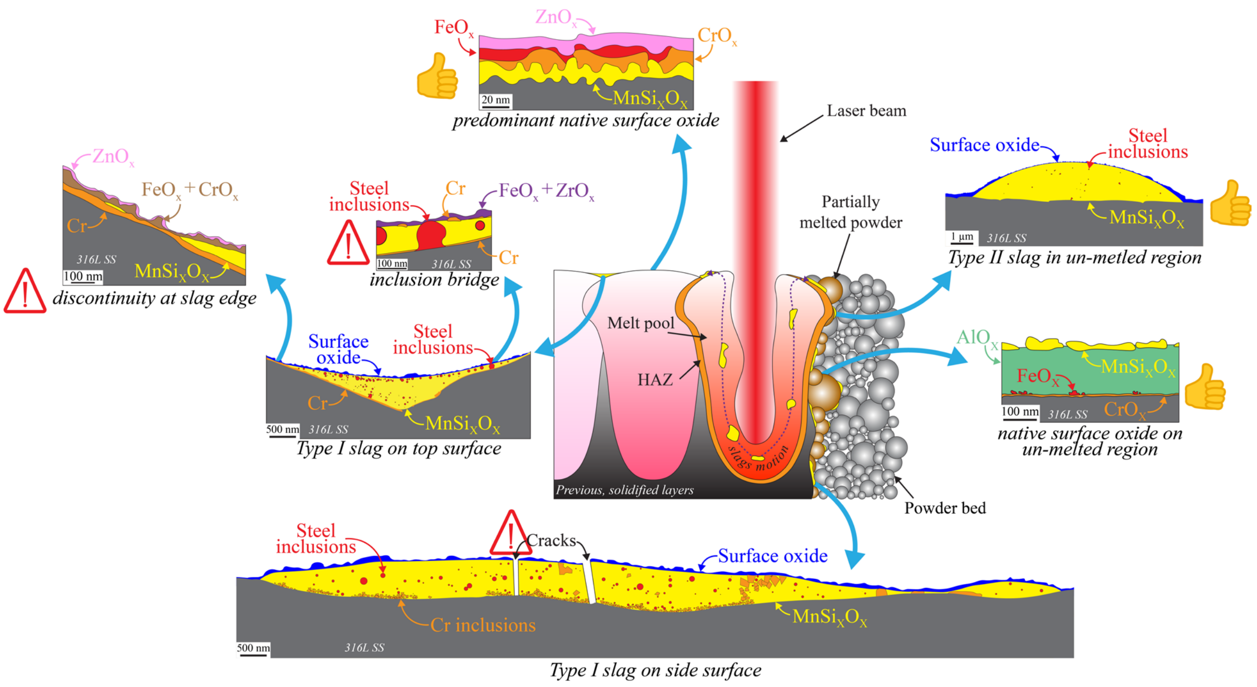

Supplementary Figure 15: Schematic summarizing composition and location of various surface features such as native surface oxides and silicate slags. All oxides schematics were made from the transmission electron microscopy images shown throughout the article. Exclamation marks highlight weak points in Type I slags. Thumb-ups mark regions at the surface that are well protected.
